# Tuberous Sclerosis Complex 1 Regulates dE2F1 Expression during Development and Cooperates with RBF1 to Control Proliferation and Survival

**DOI:** 10.1371/journal.pgen.1001071

**Published:** 2010-08-19

**Authors:** Ting-Chiu Hsieh, Brandon N. Nicolay, Maxim V. Frolov, Nam-Sung Moon

**Affiliations:** 1 Department of Biology, Developmental Biology Research Initiative, McGill University, Montreal, Canada; 2 Department of Biochemistry and Molecular Genetics, University of Illinois Chicago, Chicago, Illinois, United States of America; Fred Hutchinson Cancer Research Center, United States of America

## Abstract

Previous studies in *Drosophila melanogaster* have demonstrated that many tumor suppressor pathways impinge on Rb/E2F to regulate proliferation and survival. Here, we report that Tuberous Sclerosis Complex 1 (TSC1), a well-established tumor suppressor that regulates cell size, is an important regulator of dE2F1 during development. In eye imaginal discs, the loss of *tsc1* cooperates with *rbf1* mutations to promote ectopic S-phase and cell death. This cooperative effect between *tsc1* and *rbf1* mutations can be explained, at least in part, by the observation that TSC1 post-transcriptionally regulates dE2F1 expression. Clonal analysis revealed that the protein level of dE2F1 is increased in *tsc1* or *tsc2* mutant cells and conversely decreased in *rheb* or *dTor* mutant cells. Interestingly, while *s6k* mutations have no effect on dE2F1 expression in the wild-type background, S6k is absolutely required for the increase of dE2F1 expression in *tsc2* mutant cells. The canonical TSC/Rheb/Tor/S6k pathway is also an important determinant of dE2F1-dependent cell death, since *rheb* or *s6k* mutations suppress the developmentally regulated cell death observed in *rbf1* mutant eye discs. Our results provide evidence to suggest that dE2F1 is an important cell cycle regulator that translates the growth-promoting signal downstream of the TSC/Rheb/Tor/S6k pathway.

## Author Summary

Tuberous Sclerosis Complex genes 1 (TSC1) is a downstream component of the Insulin Receptor signaling pathway that is often deregulated in many tumors. In this study, we discovered that the fruit fly homolog of TSC1 regulates E2F transcription factor by controlling protein expression. E2F family proteins are key regulators of cellular division, and other tumor promoting events are previously shown to regulate E2F activity. Our findings demonstrate the importance of altering the E2F activity during tumorigenesis and provide new insights into the crosstalk between tumor promoting events.

## Introduction

Retinoblastoma (Rb) family proteins are important regulators of cell cycle progression and survival (reviewed in [1], [2]). Orthologs of Rb exist in all metazoans where their functions are evolutionarily conserved (reviewed in [3]). Their best-known molecular function is to physically interact with E2F family proteins and transcriptionally repress E2F-regulated target genes. Genome-wide expression studies revealed that genes involved in various biological processes, such as cell cycle progression, survival, and development, are regulated by E2F family proteins [4]–[6]. As a consequence, the loss of Rb family genes in mice results in developmental defects that are often associated with uncontrolled S-phase entry and ectopic cell death [7]–[9]. Importantly, reducing E2F activity largely suppresses the Rb mutant phenotypes, indicating that deregulated E2F activity is responsible for the defects [10], [11]. Overall, E2F family proteins are the key molecular targets of Rb family proteins and responsible for the developmental consequence of *Rb* inactivation.

The long-term consequence of Rb inactivation in mammals is tumorigenesis. In humans, the loss of Rb is believed to be a critical step for retinoblastoma development. Moreover, Rb is believed to be functionally inactivated in most, if not all, cancers (reviewed in [12]). In mice, *Rb* heterozygosity (*Rb+/−*) results in the formation of pituitary and thyroid tumors [7], [13]–[16]. The wild type copy of the *Rb* gene is lost in these tumors, illustrating the importance of *Rb* as a tumor suppressor gene. Moreover, conditional knockout of *Rb* and an additional member of the *Rb* family gene, *p107* or *p130*, in mouse retina is sufficient to promote retinoblastoma development [17]–[20]. Similar to the developmental phenotype, deregulated E2F plays a major role during tumorigenesis in *Rb* mutant mice. In a pituitary tumor model, the loss of *E2f-1* or *E2f-3* reduces the frequency of tumor development [21], [22]. More recently, the importance of E2F family proteins in human cancer is further illustrated by the findings that E2F family proteins themselves are often deregulated in many types of cancers (reviewed in [23]). Clearly, E2F family proteins play a critical role during tumorigenesis and also contribute to the developmental defects observed in Rb mutant animals.

Although it is clear that studying the function of E2F is crucial to understand the biology of Rb mutant animals and cancers, it has been difficult to dissect the *in vivo* roles of E2F family genes in mammals. One of the difficulties is the fact that E2F family proteins can functionally compensate for each other, which is particularly true for the subset of E2F proteins called “activator E2Fs” (reviewed in [24]). This is best demonstrated by a recent study showing that a single “activator E2F”, E2F-3a, is sufficient to support embryonic and post-natal development in mice, and the expression of E2F-3b or E2F-1 under the control of E2F-3a promoter can perform the role of E2F-3a [25]. This study suggests that the unique developmental functions of “activator E2Fs” are largely determined by their expression patterns and not by the differences of their protein sequences. Interestingly, *Drosophila melanogaster* has only a single “activator E2F”, dE2F1. The function of dE2F1 is evolutionarily conserved and represents the three “activator E2Fs” in mammals. dE2F1 is required for cellular proliferation and controls DNA damage-induced cell death, activities that are shared by the three “activator E2Fs” in mammals (reviewed in [3]). Since dE2F1 is the sole member carrying out the function of three E2Fs in mammals, it is possible that the regulation of dE2F1 expression is more complex and tightly controlled in flies. However, the regulatory mechanism that controls dE2F1 expression in *Drosophila* is poorly understood.

Like Rb, RBF1 is the major regulator of dE2F1 in flies. Most of the *rbf1* mutant phenotypes are believed to be due to deregulated dE2F1 and can be rescued by a hypomorphic mutant allele of *de2f1* [26]. Because of its simplicity and conserved developmental function, the *Drosophila* Rb/E2F is considered as a simplified version of mammalian Rb/E2F. Although *rbf1* mutations are not sufficient to promote tumor phenotype in *Drosophila*, recent genetic studies revealed that RBF1/dE2F1 plays a crucial role when proliferation and/or survival are compromised by various tumor-promoting mutations. For example, dE2F1 is required by *hippo* mutant cells to overcome the developmentally regulated cell cycle arrest in eye imaginal discs [27]. Moreover, dE2F1-dependent cell death limits the growth promoting effect of the *archipelago* mutations in the eye, and cooperates with low EGFR activity to promote cell death [28], [29]. Interestingly, although the *Drosophila p53* (*dp53*) does not genetically interact with *rbf1* during development, dE2F1 and p53 cooperate to promote DNA damage- induced cell death as they do in mammalian systems [30]. Overall, RBF1/dE2F1 can either promote and/or limit the proliferation of cells that carry tumor-promoting mutations in flies.

Tuberous Sclerosis Complex 1 (TSC1) is a tumor suppressor gene that is mutated in benign tumors (reviewed in [31]). The *in vivo* function of TSC1 was first identified in *Drosophila melanogaster* as a regulator of cell size and proliferation (reviewed in [32]). TSC1 is a negative regulator of the Ras Homolog Enriched in Brain (Rheb), which is an activator of Target of Rapamycin (Tor). The canonical TSC/Rheb/Tor pathway has been established as a central network governing cell size and growth regulation. Although initial reports clearly demonstrated that TSC1 inactivation perturbs the cell cycle profile, less is understood about the mechanism by which TSC1 controls the cell cycle as well as cell size. Here, we demonstrate that *tsc1* mutations cooperate with *rbf1* mutations to promote both unscheduled S-phase entry and cell death during *Drosophila* eye development. This cooperative effect between *tsc1* and *rbf1* mutations can be explained, at least in part, by the observation that dE2F1 expression is post-transcriptionally increased in *tsc1* mutant cells. A dE2F-reporter construct, PCNA-GFP, is activated in *tsc1* mutant cells, and *de2f1* mutations completely suppress the ectopic cell death observed in the *rbf1* and *tsc1* double mutant cells, indicating that dE2F1 is activated by *tsc1* mutations and required for cooperative effect between *rbf1* and *tsc1* mutations. We further demonstrate that Rheb and Tor control dE2F1 expression, and *s6k* mutations completely abolish the increase of dE2F1 expression observed in *tsc2* mutant cells. These results demonstrate that the TSC/Rheb/Tor/S6k pathway is an important regulator of dE2F1 expression during development and cooperates with RBF1 to regulate cell cycle progression and survival.

## Results

### *tsc1* and *rbf1* mutations cooperate to promote S-phase entry and cell death

**Figure 1:**
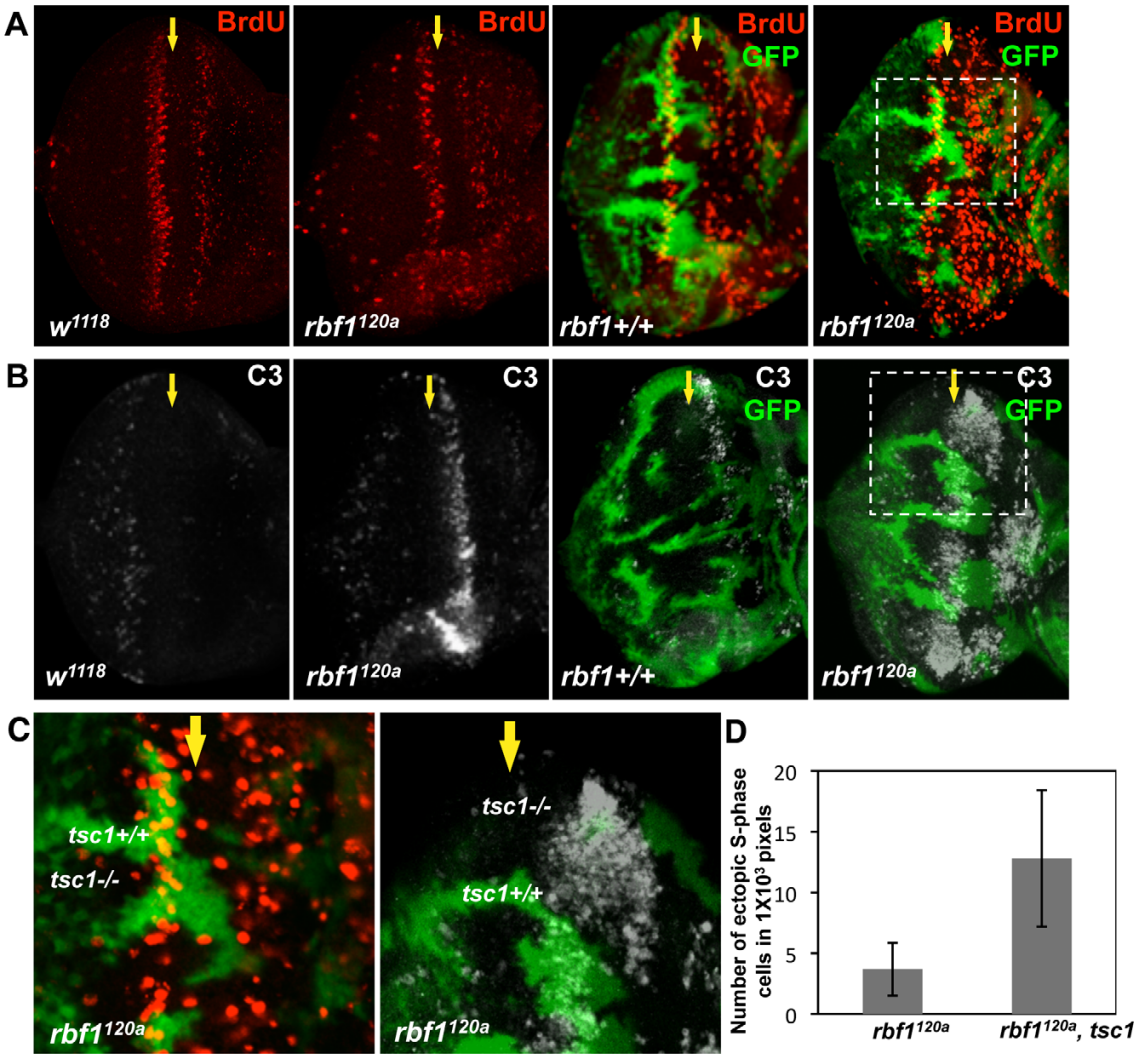
*tsc1* and *rbf1* mutations cooperate to promote S-phase entry and cell death during eye development. *tsc1^R453X^* mutant clones are generated in wild type and *rbf1^120a^* mutant eye discs by FLP-induced mitotic recombination. Wild type clones are marked with GFP (green) and the lack of GFP indicates *tsc1^R453X^* mutant clones. Control (*w^1118^*) and *rbf1^120a^* eye discs without *tsc1^R453X^* mutant clones are also presented. The position of the Morphogenetic Furrow (MF) is indicated by a yellow arrow. (A) Third instar eye discs of indicated genotypes are treated with BrdU, and S-phase cells are visualized by anti-BrdU antibody (red). (B) To visualize apoptotic cells in the eye discs of the same genotypes, antibodies that recognize the cleaved form of Caspase 3 (C3) are used (white). (C) Images of higher magnification of the eye discs containing *tsc1* mutant clones in *rbf1* mutant background are shown. Note that the cells with both *tsc1* and *rbf1* mutations ectopically enter S-phase at the MF, and the C3 staining is stronger in the double mutant clones. (D) Numbers of ectopic BrdU positive cells within the MF are counted and normalized by the sizes of clones. The clone sizes are determined by counting the numbers of pixels that encompass the region between the first and second mitotic waves. Total of 12 *rbf1* single and 20 *rbf1 tsc1* double mutant clones are analyzed. The error bars indicate standard deviation.

Ectopic S-phase entry and cell death are well-established *Rb* loss-of-function phenotypes. To address the question whether growth-promoting mutations could alter the *Rb* mutant phenotypes, we sought to determine the effects of inactivating the *Drosophila* ortholog of Tuberous Sclerosis Complex 1 (TSC1) in an *rbf1* mutant background. To test this, *tsc1* mutant clones were generated in wild type or *rbf1* mutant eye discs (Figure 1). Since homozygous *rbf1* null flies die at the first instar larval stage, we used an *rbf1* hypomorphic allele, *rbf1^120a^*. Mitotic *tsc1* mutant clones were generated by expressing Flippase (FLP) with an eye-specific driver and marked by the absence of GFP. Thus, GFP negative clones in wild type background have only *tsc1* mutations while GFP negative clones in the *rbf1^120a^* background have both *rbf1* and *tsc1* mutations. Third instar larval eye discs were dissected and immunostained with anti-BrdU antibodies. During normal eye development in *Drosophila*, S- phase cells, which can be labeled with BrdU, are found at the anterior portion of the eye imaginal disc where cells are asynchronously dividing, and immediately posterior to the Morphogenetic Furrow (MF) where some cells undergo an extra S-phase called the Second Mitotic Wave (Figure 1A). At the MF, asynchronously dividing precursor cells arrest in G1 and begin differentiation process. Therefore, normally, there is no BrdU incorporating cells at the MF. Surprisingly, in clones that are double mutant for *rbf1* and *tsc1*, ectopic S-phase cells were readily observed at the MF (Figure 1C). Since we can occasionally detect *rbf1* mutant cells entering S-phase at the MF, we compared the number of ectopic BrdU positive cells at the MF between *rbf1* single and *rbf1 tsc1* double mutant clones. We normalized the number of ectopic BrdU positive cells by the clone size, which is measured by the number of the pixels in images taken at the same magnification. Clones that do not contain ectopic BrdU positive cells are excluded from the analysis. We determined that, on average, 3.7±2.2 ectopic S-phase cells/1000 pixels are present in the *rbf1* clones while 12.4±5.6 ectopic S-phase cells/1000 pixels cells are present in the *rbf1 tsc1* double mutant clones, showing more than 3 fold increase. This result indicates that RBF1 and TSC1 cooperatively regulate G1 to S- phase transition. Next, we stained for dying cells with anti-cleaved Caspase 3 antibodies (C3). *rbf1* mutant cells undergo apoptosis at the anterior region of the MF, and this is not observed in the wild type eye disc (Figure 1B). We had previously reported that this developmentally regulated cell death in *rbf1* mutant eye discs is dE2F1-dependent [29]. *tsc1* mutant cells also undergo apoptosis just anterior to the MF though the level of cell death is much lower than what is observed in *rbf1^120a^* eye discs. However, in clones that are double mutant for *rbf1* and *tsc1*, we observed a great increase in C3 staining at the MF and the anterior region of the eye disc (Figure 1B and 1C). Therefore, we concluded that RBF1 and TSC1 synergistically promote survival as well as G1 arrest during *Drosophila* eye development.

### TSC1 regulates dE2F1 protein expression post-transcriptionally

**Figure 2:**
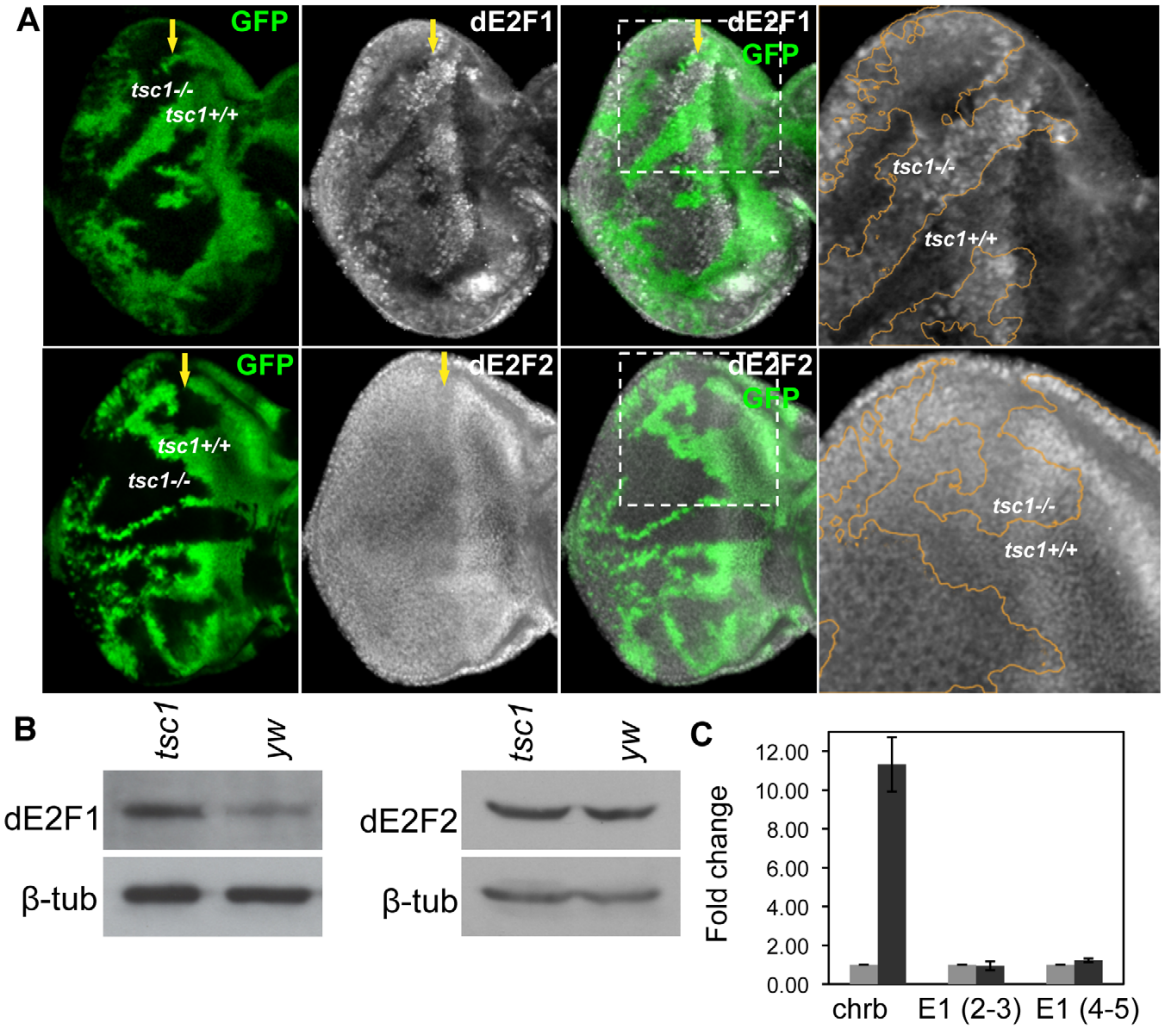
Tsc1 regulates dE2F1 protein expression post-transcriptionally. (A) *tsc1^R453X^* mutant clones are generated in the eye disc as described previously and immunostained with anti-dE2F1 or anti-dE2F2 antibodies. Images of higher magnification for the indicated areas (dotted line) are presented in the rightmost panel. The orange line indicates the clonal boundary. Note that the intensity of dE2F1 staining is stronger in *tsc1* mutant clones, whereas dE2F2 staining is unaltered. (B) The protein level of dE2F1 and dE2F2 in *tsc1* mutant eye discs is determined by immunoblot. Eye-antenna imaginal discs that are mostly comprised of *tsc1* mutant cells are used. *β-*tubulin is used as a loading control. (C) Quantitative real-time PCR is used to compare the level of *de2f1* RNA in the control (*yw*) and *tsc1* mutant eye discs. The average fold difference of three independent triplicated experiments is presented. Primers for *de2f1* were designed to span an intron, covering either the second and third exons, E1 (2–3) or the fifth and the sixth exons E1 (5–6). *charybdis* (*chrb*), whose expression is known to be upregulated by *tsc1* mutations, is used as a positive control. The error bars indicate standard deviation of the three independent experiments, ±1.40 for Chrb, ±0.23 for E1 (2–3), and ±0.10 for E1 (5–6).

RBF1 is best characterized as a regulator of dE2F1 transcription factors whose activity promotes both S-phase entry and apoptosis. Since we observed that *tsc1* mutations are able to enhance both ectopic S-phase entry and cell death phenotypes in *rbf1* mutant cells, we sought to determine if dE2F1 itself is deregulated by *tsc1* mutations. Eye discs containing *tsc1* mutant clones were generated as described previously and immunostained with an anti-dE2F1 antibody. We observed that the intensity of dE2F1 staining is clearly stronger in *tsc1* homozygous mutant clones throughout the eye disc, both in dividing and differentiating cells (Figure 2A and Figure S1A). Moreover we detected similar increase in antenna and wing discs, indicating that the effect on dE2F1 protein expression is not tissue-specific (Figure S1B and S1C). Importantly, the intensity of dE2F2 staining, the only other member of the E2F family in *Drosophila*, is unchanged in *tsc1* mutant cells (Figure 2A), indicating that the effect of *tsc1* mutations on dE2F1 expression is specific. To confirm the immunostaining result, we performed immunoblot assays using protein extracts from eye imaginal discs comprised mostly of *tsc1* mutant cells (see Materials and Methods). Consistent with the immunostaining experiments, dE2F1 protein level is higher in *tsc1* mutant eye discs than in control discs while no difference is detected in dE2F2 protein level (Figure 2B). To determine whether TSC1 regulates the level of *de2f1* RNA, we performed real-time quantitative PCR (RTq-PCR). RNA was isolated from eye discs of the same genotypes used for immunoblot. We designed *de2f1* specific primers that span an intron and amplified portions of two exons (second and third exons or fourth and fifth exons) to distinguish the PCR products from cDNA and genomic DNA. *charybdis* (*chrb*), a previously reported TSC1 regulated gene is used as a positive control [33]. Similar to the published result, we observed that the level of *chrb* RNA is increased by 11-fold in *tsc1* mutant eye discs (Figure 2C). However, we could not detect any significant changes in *de2f1* RNA level in *tsc1* mutant eye discs (Figure 2C). Therefore, we concluded that TSC1 regulates dE2F1 expression post- transcriptionally.

### Transcription of dE2F1 target genes is activated in *tsc1* mutant cells

**Figure 3:**
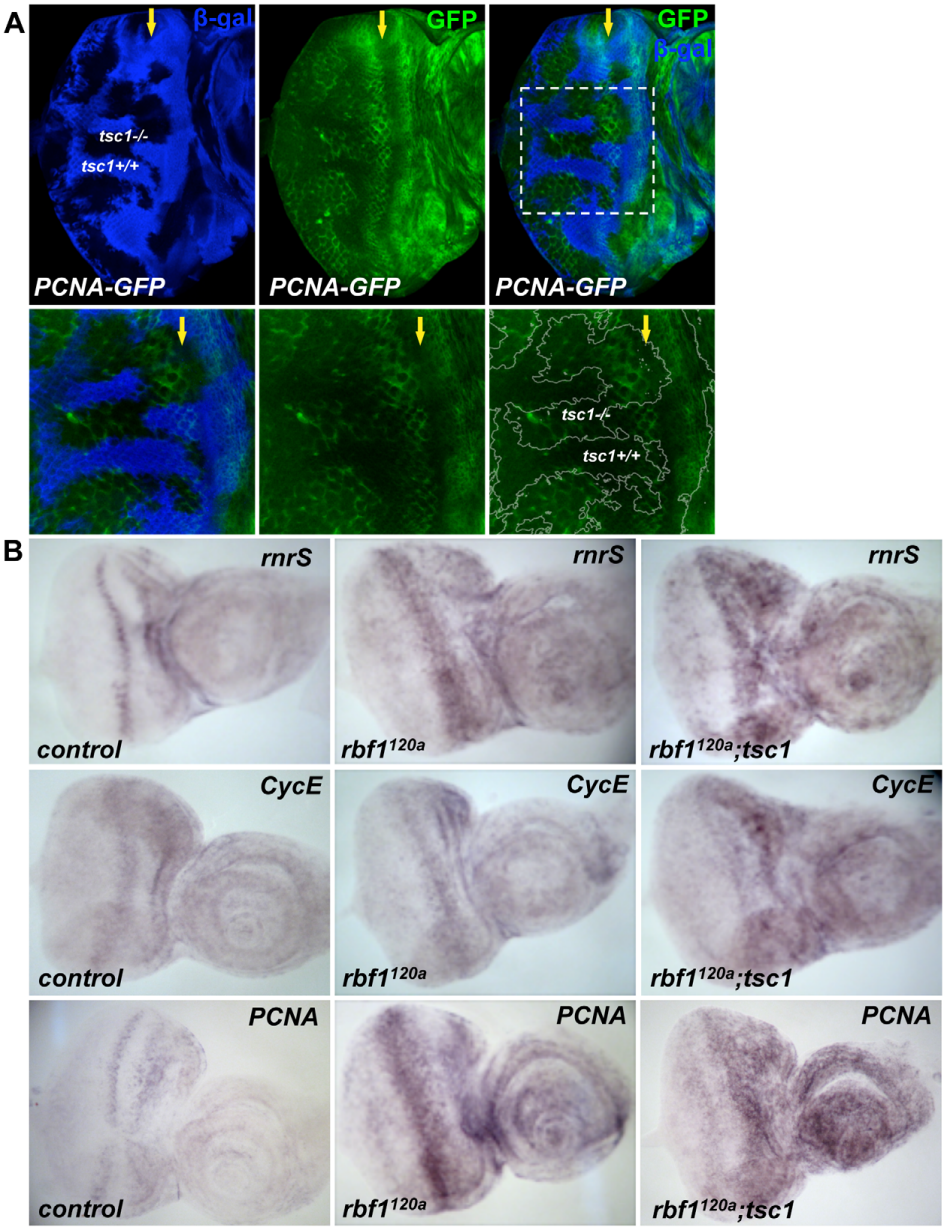
Transcription of dE2F1 target genes is activated in *tsc1* mutant cells. (A) Mitotic clones of *tsc1^R453X^* are generated in the eye disc of *PCNA-GFP* transgenic flies. *PCNA- GFP* is a reporter construct where GFP (green) is expressed under the control of the *PCNA* promoter, a known dE2F1 target. Wild type clones are marked by the presence of β- galactosidase for this experiment (blue). Note that GFP expression is increased in *tsc1* homozygous mutant clones at the posterior of the MF. Images of higher magnification of the mitotic clones are also shown (lower panel). (B) *In situ* hybridization assay is used to compare expression patterns of three dE2F1 target genes, *rnrS, CycE*, and PCNA. *rbf1 tsc1* double mutant eye discs are generated as described previously. Since *rbf1 tsc1* double mutant eye discs are generated by mitotic recombination using a recessive cell lethal mutation, the control and *rbf1* eye discs are generated by inducing mitotic recombination between the wild type FRT chromosome against the same recessive cell lethal mutation (See Materials and Methods). Note the expression of *rnrS, CycE,* and *PCNA* in *rbf1^120a^* eye discs is highest at the MF. In contrast, strong expression of *rnrS*, *CycE,* and *PCNA* is observed both at the MF and in the anterior region of the *rbf1 tsc1* double mutant eye disc.

Next, we examined whether the transcription of a dE2F1 target gene is activated in *tsc1* mutant cells. To address this question, we used a reporter construct, *PCNA-GFP*, whose GFP expression is under the control of the *PCNA* promoter, a well-established dE2F1 target gene. As shown in Figure 3, GFP expression is increased in *tsc1* mutant cells in the posterior portion of the eye disc, suggesting that, at least in this region, the increase of dE2F1 protein is sufficient to activate the transcription of a target gene. Importantly, the abnormal BrdU positive cells observed in the same region of *tsc1* mutant clones are scarcely present (Figure 1A), indicating that the increase in dE2F1-reporter activity is not an indirect consequence of ectopic S-phase cells. We also sought to determine if *tsc1* mutations could further activate dE2F1 target gene expression in *rbf1* mutant cells. Our attempt to compare dE2F1 target gene expression between *rbf1* single and *rbf1 tsc1* double mutant eye discs by RTq-PCR did not provide any conclusive results (data not shown). This was somewhat expected since a substantial number of *rbf1 tsc1* double mutant cells, presumably cells with hyperactive dE2F1, undergo cell death (Figure 1B and Figure S2A). Therefore, we decided to perform an *in situ* hybridization experiment, hoping to detect specific changes in a subset of surviving *rbf1 tsc1* double mutant cells. Expression patterns of dE2F1 target genes *(rnrS, Cyclin E*, and *PCNA*) were determined using antisense RNA probes. In wild type eye discs, the expression pattern of these target genes resembles that of BrdU staining since their transcription is activated during the G1/S phase transition (Figure 3B left panel). In *rbf1* mutant eye discs, dE2F1 target genes are strongly expressed at the MF where dE2F1 protein expression is normally high (Figure 3B middle panel). It is probable that, in *rbf1* mutant eye discs, dE2F1 target gene expression is mainly controlled by dE2F1 protein level since cell cycle-dependent regulation by RBF1 is absent. Interestingly, in *rbf1 tsc1* double mutant eye discs, dE2F1 target genes are strongly expressed both at the MF and in the anterior region of the eye disc (Figure 3B right panel). We reasoned that, since *rbf1* mutant cells at the MF already express a high level of dE2F1 protein (previously shown in [29]), there is only a small margin for dE2F1 target gene expression to be further activated by *tsc1* mutations. However, in the anterior region of the eye disc where the dE2F1 protein expression is normally kept low [29], *tsc1* mutations can have a greater effect on dE2F1 activity and target gene expression. As a consequence, dE2F1 target genes are strongly expressed both at the MF and in the anterior region of *rbf1 tsc1* double mutant eye discs, reaching the threshold of expression before undergoing cell death. Supporting this idea, ectopic cell death in *rbf1 tsc1* double mutant eye discs is mainly observed at the MF and in the anterior region of the eye disc (Figure S2A). Interestingly, we could not detect much increase in dE2F1 target gene expression in the posterior region of *rbf1 tsc1* double mutant eye discs, somewhat contradicting the result obtained by the PCNA-GFP reporter construct (Figure 3A). One explanation is that the *in situ* hybridization experiment is not as sensitive and quantitative as the PCNA-GFP reporter construct. We also found that the residual RBF1 proteins in the hypomorpic *rbf1^120a^* allele are mostly expressed in the posterior region of the MF, explaining why cells in this region do not show much an increase in dE2F1 target gene expression (Figure S2B). Nevertheless, these results indicate that *tsc1* mutations can activate dE2F1 target gene expression in the wild type and *rbf1* mutant backgrounds.

### dE2F1 is required for the ectopic cell death induced by *rbf1* and *tsc1* mutations

**Figure 4:**
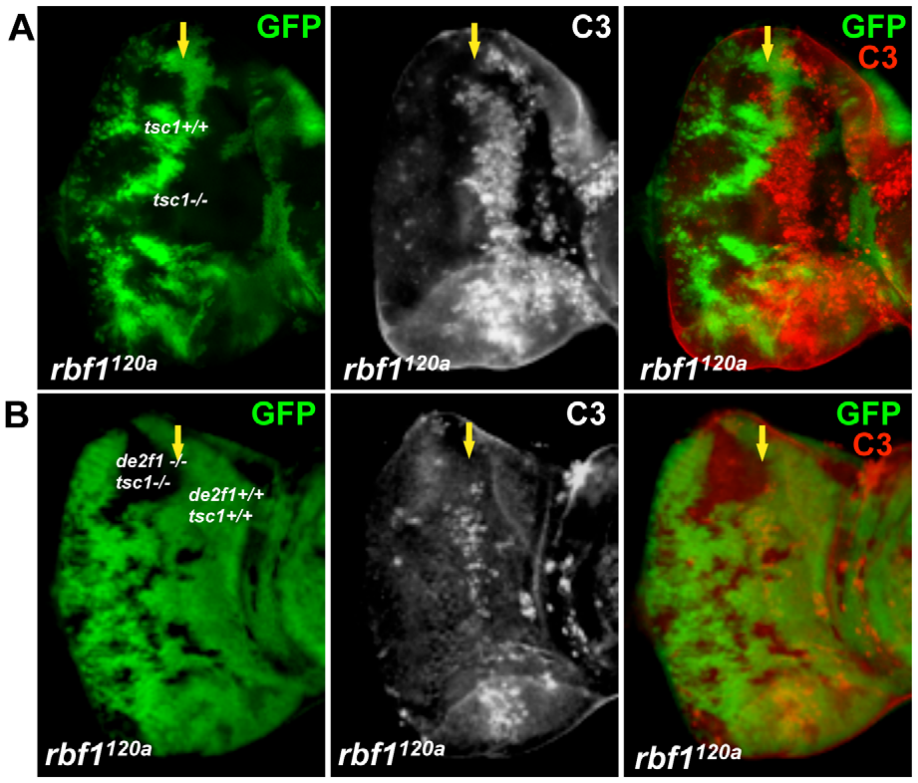
dE2F1 is required for the ectopic cell death induced by *tsc1* mutations in the *rbf1^120a^* eye discs. Mitotic clones of *tsc1^f01910^* single (A) or *tsc1^f01910^* and *de2f1^729^* double mutants (B) were generated in the *rbf1^120a^* mutant background. Wild type cells were marked with GFP (green). Apoptotic cells are visualized by immunostaining with C3. Note that the ectopic cell death induced by *tsc1* mutations in *rbf1* mutant eye discs is completely suppressed by *de2df1* mutations.

To determine if the cooperative effect on cell death by *rbf1* and *tsc1* mutations is dE2F1- dependent, we generated an allele with an FRT chromosome carrying both *tsc1* and *de2f1* mutations. For this allele, we used the *tsc1^f01910^* allele that contains a piggyBac transposable element inserted in the intron 6 of the *tsc1* locus. Generating *tsc1^f01910^* clones in *rbf1^120a^* eye discs produces a similar increase in the level of ectopic cell death observed in Figure 1 (Figure 4A). When *tsc1^f01910^* and *de2f1^729^* double mutant clones are generated in *rbf1^120a^* eye discs, we noticed that the sizes of *tsc1 de2f1* double mutant clones are much smaller than that of *tsc1* single mutant clones (compare Figure 4A and 4B). The sizes of *tsc1 de2f1* double mutant clones in the wild type background are also small (data not shown), indicating that the loss of *de2f1* severely compromises proliferation of *tsc1* mutant cells. Occasionally, we were able to obtain *rbf1^120a^* mutant eye discs with substantial sizes of the *tsc1 de2f1* double mutant clones. We performed C3 staining to measure the level of cell death in *rbf1*, *tsc1*, and *de2f1* triple mutant cells in these eye discs. Interestingly, the prevailing cell death phenotype observed in *rbf1 tsc1* double mutant cells at the MF is no longer present in *rbf1 de2f tsc1* triple mutant cells (Figure 4B). This result demonstrates that the increased level of ectopic cell death observed in *rbf1 tsc1* double mutant cells is dE2F1-dependent.

### Rheb regulates dE2F1 expression and dE2F1-dependent cell death

**Figure 5:**
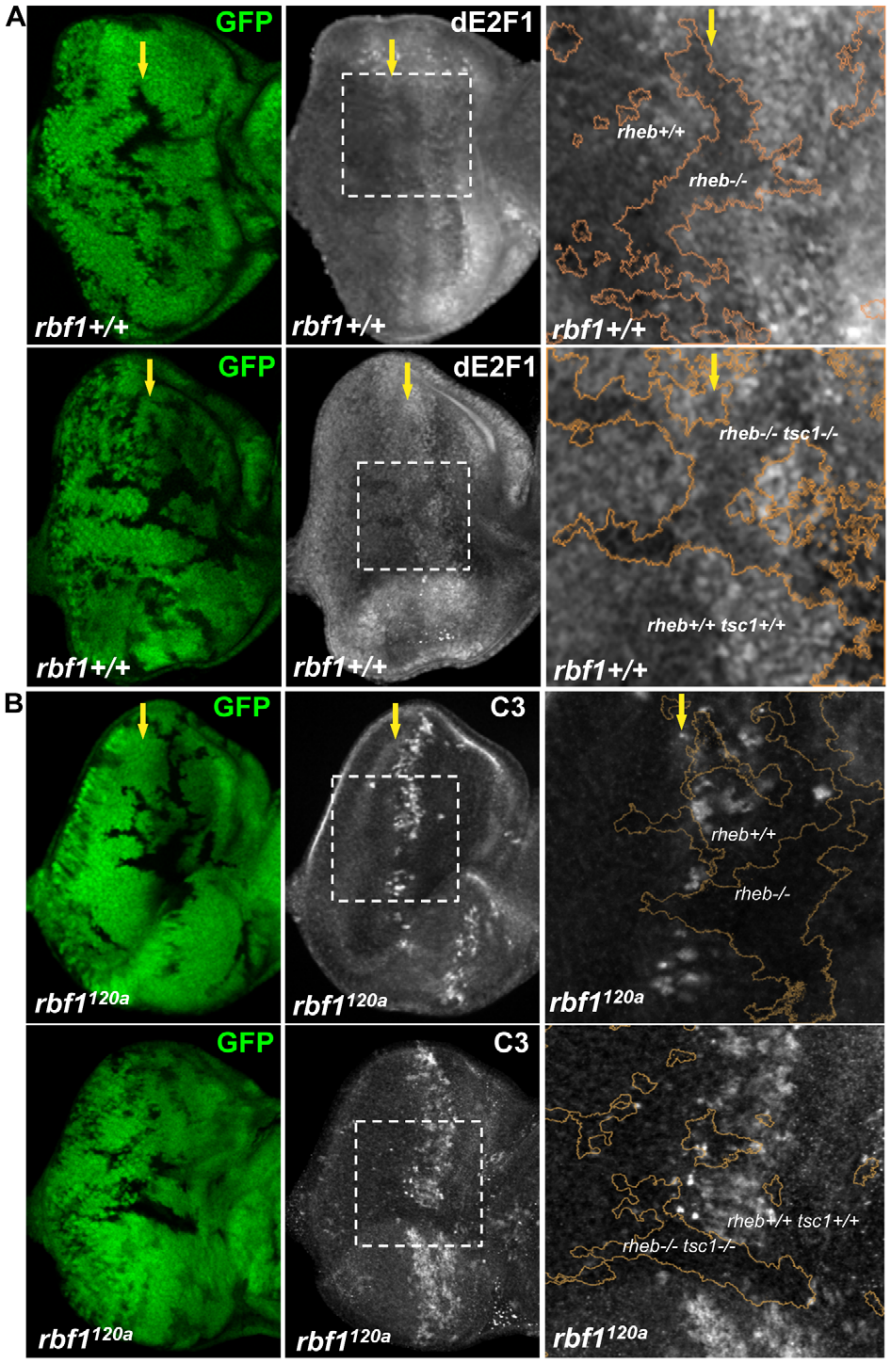
Rheb promotes dE2F1 expression during eye development and dE2F1- dependent cell death in *rbf1* mutant eye discs. (A) Mitotic clones of *rheb^2D1^* or double- mutant clones of *rheb^2D1^* and *tsc1^R453X^* are generated in the eye discs and immunostained with an anti-dE2F1 antibody (white). Images of higher magnification with outlined clonal boundaries (orange) is also shown. Note the reduced dE2F1 staining in *rheb* mutant clones. (B) *rheb^2D1^* or double-mutant clones of *rheb^2D1^* and *tsc1^R453X^* are generated in *rbf1^120a^* mutant eye discs and immunostained with C3 to visualize apoptotic cells (white). Note the discontinued stripe of cell death in *rheb* mutant clones.

Next, we asked if the known downstream regulators of TSC1 could regulate dE2F1 expression. We first determined the effect of *rheb* loss-of-function mutations on dE2F1 expression by generating mitotic mutant clones of *rheb* in the eye disc. Rheb is a Ras superfamily GTPase whose activity is negatively regulated by TSC1. As shown in Figure 5A, dE2F1 protein level is reduced, though not absent, in *rheb* mutant cells. This is best observed at the MF where dE2F1 expression is normally high [34]. We then asked if Rheb is required for the increased dE2F1 expression in *tsc1* mutant cells. dE2F1 protein level is also reduced in *tsc1 rheb* double mutant cells (Figure 5A), indicating that Rheb is an important downstream regulator of TSC1 controlling dE2F1 expression. We concluded that, although not essential, Rheb regulates dE2F1 expression during eye development, and is clearly required for dE2F1 upregulation in *tsc1* mutant cells. Since Rheb controls dE2F1 expression, we next tested if Rheb is also required for dE2F1-dependent cell death. To test this, we generated *rheb* mutant clones in the *rbf1^120a^* mutant eye disc where deregulated dE2F1 produces a stripe of apoptotic cells at the anterior region of the MF (Figure 1A and [29], [35]). As shown in Figure 5B, this stripe of cell death is interrupted by *rheb* mutant clones. Moreover, the ectopic cell death observed in *rbf1 tsc1* double mutant cells is completely suppressed by *rheb* mutations. These results indicate that Rheb is an important regulator of dE2F1-dependent cell death as well as dE2F1 expression.

### Tor, but neither S6k nor 4E-BP, is required for dE2F1 expression during *Drosophila* eye development

**Figure 6:**
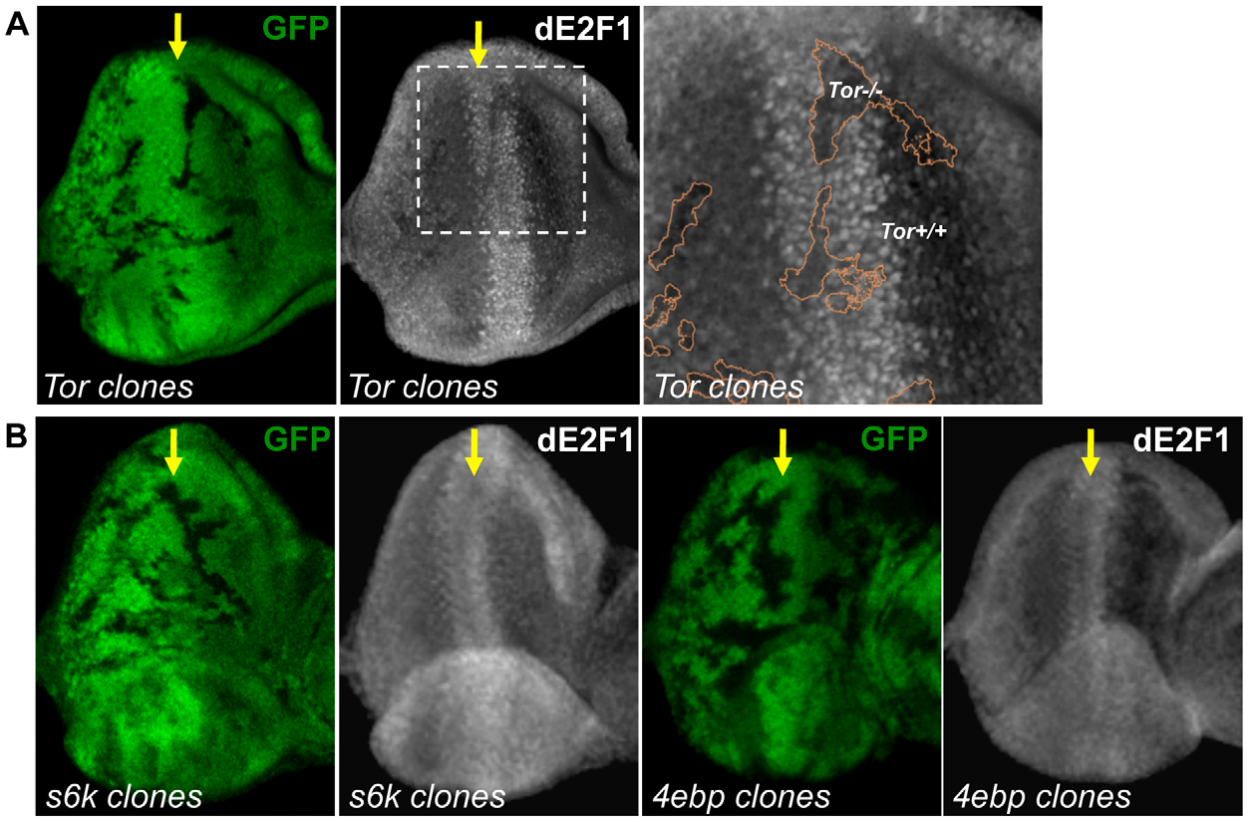
Tor is required for dE2F1 expression during eye development, but neither *s6k* or *4ebp* mutations affect dE2F1 expression. (A) Mitotic clones of *Tor^2L19^* are generated in the eye discs and immunostained with an anti-dE2F1 antibody (white). Images of higher magnification with outlined clonal boundaries (orange) are also shown. dE2F1 staining is clearly reduced in the *tor* mutant clones. (B) *s6k^ l-1^* or *4ebp^null^* mutant clones are generated as described previously. In contrast to *Tor* mutant clones, dE2F1 expression is unchanged in the mutant clones of either genotype.

Rheb activates the Tor serine/threonine kinase, which through phosphorylation, can either inhibit 4EBP or activate S6k. We examined whether these proteins downstream of Rheb also participate in dE2F1 regulation. To address this question, *Tor*, *s6k*, and *4ebp* mutant clones were generated in the eye disc. Similar to what is observed in *rheb* mutant clones, dE2F1 expression is reduced, but not absent, in *Tor* mutant clones, indicating that Tor participates in regulating dE2F1 expression during eye development (Figure 6A). Importantly, dE2F2 expression is unchanged in *Tor* mutant clones (data not shown). Based on this observation, we had hypothesized that dE2F1 expression levels would decrease in *s6k* mutant clones and/or increase in *4ebp* mutant clones. Surprisingly, dE2F1 expression is unchanged in either *4ebp* or *s6k* mutant clones (Figure 6B). These results suggest that Tor is required for proper dE2F1 expression during eye development while 4EBP and S6k are dispensable.

### S6k is required for the effect of TSC inactivation on dE2F1 expression and dE2F1- dependent cell death

**Figure 7:**
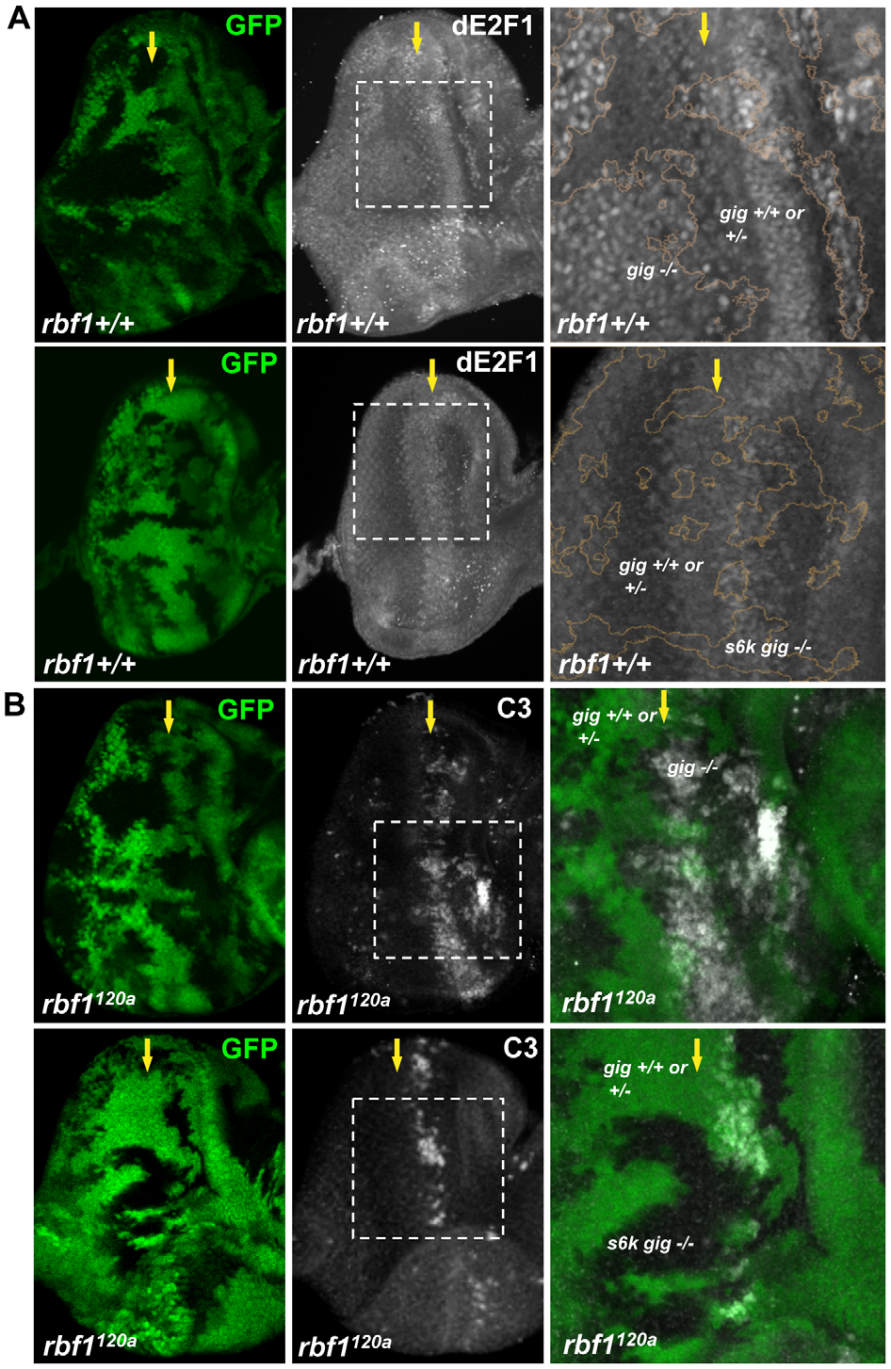
S6K is required for the effect of TSC inactivation on dE2F1 expression and dE2F1-dependent cell death in *rbf1^120a^* eye discs. (A) Mitotic clones of *gig^192^* single or *s6k^l-1^* and *gig^192^* double mutant clones are generated in the eye discs. The intensity of the GFP indicates that GFP expressing clones are composed of two genotypes, wild type and heterozygous mutations (eg. *gig+/+* or *gig+/−*). An anti-dE2F1 antibody (white) is used to determine the expression pattern of dE2F1. Images of higher magnification with outlined clonal boundaries (orange) are also presented. Note that dE2F1 expression is unchanged in *s6k gig* double mutant clones contrary to *gig* single mutant clones where dE2F1 level is clearly elevated. (B) *gig^192^* single or *s6k^ l-1^* and *gig^192^* double mutant clones are generated in *rbf1^120a^* mutant eye discs and stained with C3 to visualize apoptotic cells. The increased level of apoptosis by *gig* mutations is suppressed by *s6k* mutations.

The fact that the loss of neither *4ebp* nor *s6k* has an effect on dE2F1 expression might indicate a functional redundancy between the two genes. Alternatively, an unknown factor downstream of Tor might regulate dE2F1 expression during development. Nevertheless, we assessed whether S6k is required for the increase of dE2F1 expression observed when TSC1 is inactivated. We aimed to generate mitotic clones that are double mutants for *tsc1* and *s6k*. However, because *tsc1* and *s6k* are on the opposite arms of the third chromosome, we used a mutant allele of *tsc2* (or *gig* in *Drosophila*), which is on the same chromosomal arm as *s6k*. TSC1 and TSC2 function together as a heterodimer, and mutations of *tsc1* or *tsc2* yield very similar phenotypes [36]–[38]. As expected, dE2F1 expression is elevated in *gig* mutant clones (Figure 7A). Furthermore, similar to what was observed in *tsc1* mutant clones in the *rbf1^120a^* mutant background, the level of ectopic cell death was increased in *gig* mutant clones generated in *rbf1^120a^* mutant eye discs (Figure 7B). Surprisingly, the effects of *gig* mutations on dE2F1 expression and ectopic cell death are completely suppressed by *s6k* loss-of-function mutations. We observed that the level of dE2F1 expression in *s6k gig* double mutant clones is unchanged compared to the control (Figure 7A), and the ectopic cell death observed in *rbf1 gig* double mutant cells is completely absent in *rbf1 gig s6k* triple mutant cells (Figure 7B). Moreover, we observed that the basal level of dE2F1-dependent cell death normally present in the *rbf1^120a^* mutant eye disc (the stripe of cell death, Figure 1B) is also suppressed (Figure 7B). These results indicate that *s6k* is required for both the elevation of dE2F1 expression upon TSC inactivation and the increased level of cell death in *rbf1 gig* double mutant cells. In summary, our genetic studies led us to conclude that TSC1 and TSC2 regulate dE2F1 expression and dE2F1-dependent cell death via the canonical Rheb/Tor/S6k pathway during *Drosophila* eye development.

## Discussion

The loss of Rb leads to hyperactivation of E2F family proteins, which is a crucial event during tumorigenesis. Here, we demonstrate that the *Drosophila* ortholog of TSC1 tumor suppressor cooperates with RBF1 to regulate dE2F1 activity during development. TSC1 post- transcriptionally regulates dE2F1 expression, and the loss of *tsc1* cooperates with *rbf1* mutations to promote unscheduled S-phase entry and cell death. This effect of *tsc1* mutations on dE2F1 expression requires the components of canonical TSC/Rheb/Tor pathway that are major regulators of cellular growth. Our study provides evidence to suggest that dE2F1 is an important protein that couples growth signals to cell cycle progression.

Recent studies have identified that pro-proliferative and pro-apoptotic activities of dE2F1 are engaged by various *Drosophila* tumor suppressor genes, such as *hippo* and *archipelago* [27], [28]. Our findings add *tsc1/2* tumor suppressor genes to this list. Previously, dE2F1 or Cyclin E overexpression is shown to bypass starvation induced G1 arrest at least in endoreduplicating tissues [39]. Moreover, similar to dE2F1, expression of Cyclin E is elevated in *tsc1* mutant cells in eye imaginal discs. [36]–[38]. Perhaps, restricting the expression of cell cycle regulators, such as dE2F1 and Cycline E, is a part of the molecular mechanisms by which nutrient deprivation induces G1 arrest. Interestingly, overexpression of dE2F1 or Cycline E does not overcome starvation-induced G1 arrest in larval neuroblasts, indicating that, in mitotic cells, neither dE2F1 nor Cycline E is the limiting factor [39]. Consistent with this observation, we could not observe any appreciable increase in the size of *rheb* or *Tor* mutant clones in *rbf1* mutant background, suggesting that multiple factors contribute to the proliferative defect observed in *rheb* or *Tor* mutant cells in imaginal discs.

Interestingly, despite the elevated level of dE2F1 and Cyclin E, *tsc1* mutant clones have relatively normal patterns of BrdU staining at the MF and a limited amount of ectopic cell death. We believe that the activity of dE2F1 in *tsc1* mutant cells is normally restricted by the presence of RBF1. The fact that the increase in ectopic S-phase entry and apoptosis by *tsc1* mutations can be only observed in the *rbf1* mutant background supports this idea. We propose that the TSC/Rheb/Tor pathway during development modulates the amount of dE2F1 needed for cellular division in proportion to the cell size. Supporting this idea, previous studies have demonstrated that *tsc1* or *tsc2* mutant cells spend less time in G1, a phenotype commonly observed in cells with elevated dE2F1 activity [36]–[38], [40]. It is conceivable that the elevated level of dE2F1 proteins in *tsc1* or *tsc2* mutant cells allows them to go through G1 to S-phase transition faster where RBF1 is normally inactivated by Cyclin Dependent Kinases (CDKs).

Despite being the only “activator E2F” in *Drosophila*, it is still unclear how dE2F1 expression is regulated during development. A recent study reported that Cul4(Cdt2) E3 ubiquitin ligase mediates destruction of dE2F1 in S-phase, a mechanism that regulates dE2F1 expression in a cell cycle dependent manner [41]. Our findings here suggest that the expression of dE2F1 is also regulated by a growth-controlling network. However, at this point, we do not know the exact molecular mechanism by which dE2F1 protein level is post- transcriptionally controlled by the TSC/Rheb/Tor pathway. The finding that S6k is involved in this process supports the idea of translational control since S6k directly phosphorylates and regulates proteins involved in translation, such as RpS6, eIF4B, and eEF2K to list a few (reviewed in [42]). However, it is also equally possible that the TSC/Rheb/Tor pathway controls dE2F1 protein stability. In S2 cells, neither *tsc1* RNAi nor Rapamycin (*Tor* inhibitor) treatment in S2 cells had the same effect on dE2F1 expression observed in imaginal discs (Figure S3). It is probable that S2 cells lack factors necessary for dE2F1 regulation that are present *in vivo*. Nevertheless, it is important to note that this effect on dE2F1 expression is specific since dE2F2 expression is unchanged in *tsc1*, *rheb* or *Tor* mutant cells (Figure 2A and data not shown). Curiously, the requirement of S6k to regulate dE2F1 is limited to the context in which TSC is inactivated. The loss of *s6k* in the wild type background has no effect on dE2F1 expression while *rheb* or *Tor* mutations reduce the level of dE2F1 proteins in the eye disc (Figure 5A and Figure 6). In mammals, it has been demonstrated that the translation of specific mRNA can be mTor-dependent but not S6k- dependent [43]. The molecular mechanism in which S6k promotes dE2F1 expression only when TSC is inactivated is presently unclear and warrants further investigation.

Another interesting finding from our study is that *s6k* mutations suppress the dE2F1-dependent cell death normally present in *rbf1* mutant eye discs (Figure 7). *s6k* mutations alone did not alter the dE2F1 expression level at least in the wild type background. Although it is not formally tested, this raises a possibility that the TSC/Tor/S6k pathway controls dE2F1-dependent cell death without altering dE2F1 expression. Interestingly, the crosstalk between the InR/Tor and the EGFR signaling pathways during *Drosophila* eye development has been recently established [44]. InR/Tor signaling regulates the timing of neuronal differentiation in the eye disc by modulating EGFR activity. Since the EGFR pathway is an important determinant of dE2F1-dependent cell death [29], S6k might promote dE2F1-dependent cell death by modulating the EGFR pathway. We speculate that the cooperative effect between *tsc1* and *rbf1* mutations is the consequence of multiple changes that include the increase in dE2F1 expression.

In cancer cells, it is generally thought that the loss of Rb function is the most common mechanism of deregulating E2F activity. However, in some types of cancers, amplification of E2F genes or overexpression of E2F family proteins have been observed (reviewed in [23]). Moreover, in a subtype of human retinoblastoma where Rb is already deficient, E2f-3 proteins are also overexpressed [45]. These observations suggest that E2F family genes themselves can be directly targeted and deregulated during tumorigenesis. It will be interesting to investigate if TSC1/2 or other tumor suppressors and oncogenes regulate the expression of E2F family proteins to promote tumorigenesis.

## Materials and Methods

### Fly stocks

All crosses have been performed at 25°C. The *rbf1* mutant allele, *rbf1^120a^*, and *de2f1* allele, *de2f1^729^*, are described previously [15], [16]. The *tsc1* alleles used in this study are *tsc1^R453X^*, a gift from Dr. Hariharan [38], and *tsc1^f01910^* (Exelixis collection, Harvard Medical School). The mutant alleles of the TSC/Rheb/Tor pathway used in this study are as follows: *Tor^2L19^ FRT40A* and *4ebp^null^* are gifts from P. Lasko [46], [47]. *s6k^ l-1^ FRT80B* is a gift from D.J. Pan [48]. The *gig^56^ FRT80B*, *FRT82B rheb^2D1^*, and *s6k^l-1^ gig^192^FRT80B* alleles were kindly provided by J.M. Bateman [44]. The *4ebp^null^ FRT40A*, *FRT82B de2f1^729^ tsc1^f01910^*, and *FRT82B rheb^2D1^ tsc1^R453X^* alleles were generated by meiotic recombination. For the double mutant alleles, presence of both mutations is verified by genetic complementation tests using multiple mutant alleles. For example, presence of both *s6k* and *gig* mutations in *s6k^ l-1^gig^192^ FRT80B* alleles were verified by crossing the alleles to *gig^52^, gig^192^*, *s6k^ l-1^* and *s6k^p{PZ}07084^* alleles individually.

### Clonal analysis

Flippase (FLP) was expressed from the *eyeless* promoter to generate mitotic clones in the eye. To examine clones in *rbf1* mutant animals, the X chromosome carrying *rbf1^120a^* and an *ey-FLP* transgene was used. Followings are the full genotypes of larvae analysed.

### Mutant clones in the wild-type background

*y w eyFlp/+ or Y; FRT82B GFP^ubi^/FRT82B tsc1^R453X^*

*y w eyFlp/+ or Y; FRT82B GFP^ubi^/FRT82B rheb^2D1^*

*y w eyFlp/+ or Y; FRT82B GFP^ubi^/FRT82B rheb^2D1^ tsc1^R453X^*

*y w eyFlp/+ or Y; GFP^ubi^ FRT40A/Tor^2L19^ FRT40A*

*y w eyFlp/+ or Y; GFP^ubi^ FRT80B/s6k^ l-1^ FRT80B*

*y w eyFlp/+ or Y; GFP^ubi^ FRT40A/4ebp^null^ FRT40A*

*y w eyFlp/+ or Y; GFP^ubi^ FRT80B/gig^56^ FRT80B*

*y w eyFlp/+ or Y; GFP^ubi^ FRT80B/s6k^l-1^ gig^192^ FRT80B*

### Mutant clones in the *rbf1^120a^* background

*w rbf1^120a^ eyFlp/Y; FRT82B GFP^ubi^/FRT82B tsc1^R453X^*

*w rbf1^120a^ eyFlp/Y; FRT82B GFP^ubi^/FRT82B tsc1^f01910^*

*w rbf1^120a^ eyFlp/Y; FRT82B GFP^ubi^/FRT82B de2f1^729^ tsc1^f01910^*

*w rbf1^120a^ eyFlp/Y; FRT82B GFP^ubi^/FRT82B rheb^2D1^*

*w rbf1^120a^ eyFlp/Y; FRT82B GFP^ubi^/FRT82B rheb^2D1^ tsc1^R453X^*

*w rbf1^120a^ eyFlp/Y; GFP^ubi^ FRT80B/gig^56^ FRT80B*

*w rbf1^120a^ eyFlp/Y; GFP^ubi^ FRT80B/s6k^l-1^ gig^192^ FRT80B*

### Immunoblot, real-time quantitative PCR, and *in situ* hybridization

*y w eyFlp/Y; FRT82B [W+] l(3)cl-R3/FRT82B (controls)*

*y w eyFlp/Y; FRT82B [W+] l(3)cl-R3/FRT82B tsc1^R453X^*

*w rbf1^120a^ eyFlp/Y; [W+] l(3)cl-R3/FRT82B*

*w rbf1^120a^ eyFlp/Y; [W+] l(3)cl-R3/FRT82B tsc1^R453X^*

### PCNA-GFP in tsc1 mutant clones

*y w eyFlp/PCNA-GFP; FRT82B LacZ^arm^/FRT82B tsc1^R453X^*

### Immunostaining and microscopy

The antibodies used in this study are: anti-dE2F1 (1/1000) [29], anti-dE2F2 (1/1000) [34], anti-RBF1 (1/100) from Dyson Lab, anti-C3 (1/200, Cell Signaling), anti-GFP-FITC (1/200, abcam), anti-β-galactosidase (Developmental Studies Hybridoma Banks [DSHB]), and anti- ELAV (DSHB). For immunostaining, third-instar eye discs were fixed in 4% formaldehyde for 20 minutes at room temperature (eye discs immunostained for anti-dE2F1 were fixed at 4°C for 30 minutes) and washed twice with 0.3% PBST (0.3% Triton X-100 in PBS) and once with 0.1% PBST (0.1% Triton X-100 in PBS). Fixed eye discs were incubated in primary antibody with 0.1% PBST and 5% normal goat serum (NGS) at room temperature for 3 hours. After four washes with 0.1% PBST, eye discs were incubated in secondary antibody with 0.3% PBST and 5% NGS at room temperature for 2 hours. Immunostained eye discs were then washed five times with 0.1% PBST at room temperature and mounted for confocal microscopy imaging (Zeiss LSM).

### *In situ* hybridization

For *in situ* hybridization experiments, eye-antennal discs were prepared as described previously [26]. Anti-sense RNA probes were generated using cDNA clones LD41588, LD17578, and LD45889 for *rnrS, CycE,* and *PCNA* respectively. After hybridization, Alkaline Phosphatase conjugated anti-DIG antibodies were used to detect DIG labeled anti- sense RNA probes. For each target genes, more than 20 eye antennal discs were analyzed and the representative images were chosen to be presented.

### Immunoblotting

40 eye discs of *tsc1* mutant and control animals were dissected and used for Western blot as previously described [29].

### Real-Time Reverse Transcriptase PCR

The average of three independent experiments of triplicate-PCR reaction is presented. Total RNA was isolated from 40 eye-antenna eye discs with RNeasy Mini kit (QIAGEN) according to manufacturer's protocol, and reverse transcribed using DyNAmo cDNA Synthesis Kit (Finnzymes) according to manufacturer's instructions. Quantitative PCR reactions were performed with DyNAmo Flash SYBR Green qPCR Kit (Finnzymes). Quantification was determined by comparative threshold cycle method (CT) on Bio-Rad CFX Manager software. Both *rp49* and *β-*tubulin were used as normalization controls in a single experiment. All primers were designed with Primer3 (Whitehead Institute fozr Biomedical Research primer3 shareware [http://frodo.wi.mit.edu/primer3/]). Primer pairs used are:

chrb1F (AACTGCAGGCTCAGCTACG)

chrb1R (CGCTCTCGAACTCAATGAAG)

de2f12-3F (CAGCACCACCACCAAAATC)

de2f12-3R (ACTGCTAGCCGTATGCTTCTG)

de2f15-6F (TACAGCCATGACCGCAAC)

de2f15-6R (GTTCAGCGCATACGGATAGTC)

tubulin-F (ACATCCCGCCCCGTGGTC)

tubulin-R (AGAAAGCCTTGCGCCTGAACATAG)

Rp49-F (TACAGGCCCAAGATCGTGAAG)

Rp49-R (GACGCACTCTGTTGTCGATACC)

## Supporting Information

Figure S1 Tsc1 regulates dE2F1 protein level both in proliferating and differentiating cells in imaginal discs. (A) *tsc1^R453X^* mutant clones are generated in the eye- antenna disc as previously described and immunostained with an anti-dE2F1 antibody. Images at two different focal planes of a single eye disc are shown. The upper panel shows increased expression of dE2F1 proteins in *tsc1* mutant clones at the anterior region of the eye disc. The lower panel shows increased dE2F1 expression in *tsc1* mutant clone at the posterior region of the eye disc. The magnified views of indicated area are also presented. (B) An antenna disc that contains *tsc1^R453X^* mutant clones is shown. As in eye imaginal discs, dE2F1 expression is increased in tsc1 mutant clones. (C) *tsc1^R453X^* mutant clones are generated in the wing disc using heat shock driven Flippase. Presumptive notum area of the wing disc is shown. Note the increased level of dE2F1 proteins in *tsc1* mutant clones.
(3.18 MB TIF)

Figure S2 The pattern of ectopic cell death in eye imaginal discs that are mostly composed of *rbf1 tsc1* double mutant cells. (A) The patterns of cell death between an *rbf1^120a^* eye disc and two eye discs carrying both *rbf1^120a^* and *tsc1^R453X^* mutations are shown (see Materials and Methods). Apoptotic cells are visualized by the C3 antibody. A dramatic increase in C3 staining is observed at the MF and in the anterior region of the eye discs carrying both *rbf1* and *tsc1* mutations. (B) *rbf1^120a^* mutant clones, marked by absence of GFP, are generated in the eye discs. Note the weak but visible RBF1 staining in *rbf1^120a^* mutant clones in the region posterior to the MF (yellow asterisk).
(1.38 MB TIF)

Figure S3 Inactivation of *tsc1* nor *Tor* affects dE2F1 protein level in S2 *Drosophila* tissue culture cells. (A) S2 cells are treated with either *white* or *tsc1* double strand RNA for 4 days and dE2F1 protein levels are measured by immunoblot. The antibody that recognizes the phospho-specific form of S6k (Cell Signaling, Cat#. 9206) is used to monitor the effect of *tsc1* depletion and anti-β-tubulin antibodies are used for loading control. Three independent experimental results are presented. (B) S2 cells are treated with DMSO or DMSO containing Rapamycin (the final concentration of 20 nM). After 16 hours of treatment, dE2F1 protein levels are measured by immunoblot. A phospho-specific S6k antibody is used to monitor the effect of Rapamycin treatment. For each lane, an equal amount of protein extract is loaded 28. (C) S2 cells are treated as described in (B). However, the amount of protein extract loaded in each lane is normalized by cell number and not by protein concentration. Note that S2 cells do not recapitulate the effect observed in imaginal discs.
(0.22 MB TIF)

## References

[1] Dyson N (1998). The regulation of E2F by pRB-family proteins.. Genes Dev.

[2] Polager S, Ginsberg D (2008). E2F - at the crossroads of life and death.. Trends Cell Biol.

[3] van den Heuvel S, Dyson NJ (2008). Conserved functions of the pRB and E2F families.. Nat Rev Mol Cell Biol.

[4] Muller H, Bracken AP, Vernell R, Moroni MC, Christians F (2001). E2Fs regulate the expression of genes involved in differentiation, development, proliferation, and apoptosis.. Genes Dev.

[5] Dimova DK, Stevaux O, Frolov MV, Dyson NJ (2003). Cell cycle-dependent and cell cycle- independent control of transcription by the Drosophila E2F/RB pathway.. Genes Dev.

[6] Ishida S, Huang E, Zuzan H, Spang R, Leone G (2001). Role for E2F in control of both DNA replication and mitotic functions as revealed from DNA microarray analysis.. Mol Cell Biol.

[7] Jacks T, Fazeli A, Schmitt EM, Bronson RT, Goodell MA (1992). Effects of an Rb mutation in the mouse.. Nature.

[8] Clarke AR, Maandag ER, van Roon M, van der Lugt NM, van der Valk M (1992). Requirement for a functional Rb-1 gene in murine development.. Nature.

[9] Lee EY, Chang CY, Hu N, Wang YC, Lai CC (1992). Mice deficient for Rb are nonviable and show defects in neurogenesis and haematopoiesis.. Nature.

[10] Tsai KY, Hu Y, Macleod KF, Crowley D, Yamasaki L (1998). Mutation of E2f-1 suppresses apoptosis and inappropriate S phase entry and extends survival of Rb- deficient mouse embryos.. Mol Cell.

[11] Ziebold U, Reza T, Caron A, Lees JA (2001). E2F3 contributes both to the inappropriate proliferation and to the apoptosis arising in Rb mutant embryos.. Genes Dev.

[12] Sherr CJ (1996). Cancer cell cycles.. Science.

[13] Harrison DJ, Hooper ML, Armstrong JF, Clarke AR (1995). Effects of heterozygosity for the Rb-1t19neo allele in the mouse.. Oncogene.

[14] Hu N, Gutsmann A, Herbert DC, Bradley A, Lee WH (1994). Heterozygous Rb-1 delta 20/+mice are predisposed to tumors of the pituitary gland with a nearly complete penetrance.. Oncogene.

[15] Du W, Dyson N (1999). The role of RBF in the introduction of G1 regulation during Drosophila embryogenesis.. EMBO J.

[16] Duronio RJ, O'Farrell PH, Xie JE, Brook A, Dyson N (1995). The transcription factor E2F is required for S phase during Drosophila embryogenesis.. Genes Dev.

[17] MacPherson D, Sage J, Kim T, Ho D, McLaughlin ME (2004). Cell type-specific effects of Rb deletion in the murine retina.. Genes Dev.

[18] Robanus-Maandag E, Dekker M, van der Valk M, Carrozza ML, Jeanny JC (1998). p107 is a suppressor of retinoblastoma development in pRb-deficient mice.. Genes Dev.

[19] Chen D, Livne-bar I, Vanderluit JL, Slack RS, Agochiya M (2004). Cell-specific effects of RB or RB/p107 loss on retinal development implicate an intrinsically death- resistant cell-of-origin in retinoblastoma.. Cancer Cell.

[20] Zhang J, Schweers B, Dyer MA (2004). The first knockout mouse model of retinoblastoma.. Cell Cycle.

[21] Yamasaki L, Bronson R, Williams BO, Dyson NJ, Harlow E (1998). Loss of E2F-1 reduces tumorigenesis and extends the lifespan of Rb1(+/−)mice.. Nat Genet.

[22] Ziebold U, Lee EY, Bronson RT, Lees JA (2003). E2F3 loss has opposing effects on different pRB-deficient tumors, resulting in suppression of pituitary tumors but metastasis of medullary thyroid carcinomas.. Mol Cell Biol.

[23] Chen HZ, Tsai SY, Leone G (2009). Emerging roles of E2Fs in cancer: an exit from cell cycle control.. Nat Rev Cancer.

[24] Stevaux O, Dyson NJ (2002). A revised picture of the E2F transcriptional network and RB function.. Curr Opin Cell Biol.

[25] Tsai SY, Opavsky R, Sharma N, Wu L, Naidu S (2008). Mouse development with a single E2F activator.. Nature.

[26] Du W (2000). Suppression of the rbf null mutants by a de2f1 allele that lacks transactivation domain.. Development.

[27] Nicolay BN, Frolov MV (2008). Context-dependent requirement for dE2F during oncogenic proliferation.. PLoS Genet.

[28] Nicholson SC, Gilbert MM, Nicolay BN, Frolov MV, Moberg KH (2009). The archipelago tumor suppressor gene limits rb/e2f-regulated apoptosis in developing Drosophila tissues.. Curr Biol.

[29] Moon NS, Di Stefano L, Dyson N (2006). A gradient of epidermal growth factor receptor signaling determines the sensitivity of rbf1 mutant cells to E2F-dependent apoptosis.. Mol Cell Biol.

[30] Moon NS, Di Stefano L, Morris EJ, Patel R, White K (2008). E2F and p53 induce apoptosis independently during Drosophila development but intersect in the context of DNA damage.. PLoS Genet.

[31] Inoki K, Corradetti MN, Guan KL (2005). Dysregulation of the TSC-mTOR pathway in human disease.. Nat Genet.

[32] Pan D, Dong J, Zhang Y, Gao X (2004). Tuberous sclerosis complex: from Drosophila to human disease.. Trends Cell Biol.

[33] Harvey KF, Mattila J, Sofer A, Bennett FC, Ramsey MR (2008). FOXO-regulated transcription restricts overgrowth of Tsc mutant organs.. J Cell Biol.

[34] Frolov MV, Moon NS, Dyson NJ (2005). dDP is needed for normal cell proliferation.. Mol Cell Biol.

[35] Tanaka-Matakatsu M, Xu J, Cheng L, Du W (2009). Regulation of apoptosis of rbf mutant cells during Drosophila development.. Dev Biol.

[36] Gao X, Pan D (2001). TSC1 and TSC2 tumor suppressors antagonize insulin signaling in cell growth.. Genes Dev.

[37] Potter CJ, Huang H, Xu T (2001). Drosophila Tsc1 functions with Tsc2 to antagonize insulin signaling in regulating cell growth, cell proliferation, and organ size.. Cell.

[38] Tapon N, Ito N, Dickson BJ, Treisman JE, Hariharan IK (2001). The Drosophila tuberous sclerosis complex gene homologs restrict cell growth and cell proliferation.. Cell.

[39] Britton JS, Edgar BA (1998). Environmental control of the cell cycle in Drosophila: nutrition activates mitotic and endoreplicative cells by distinct mechanisms.. Development.

[40] Neufeld TP, de la Cruz AF, Johnston LA, Edgar BA (1998). Coordination of growth and cell division in the Drosophila wing.. Cell.

[41] Shibutani ST, de la Cruz AF, Tran V, Turbyfill WJ, Reis T (2008). Intrinsic negative cell cycle regulation provided by PIP box- and Cul4Cdt2-mediated destruction of E2f1 during S phase.. Dev Cell.

[42] Ruvinsky I, Meyuhas O (2006). Ribosomal protein S6 phosphorylation: from protein synthesis to cell size.. Trends Biochem Sci.

[43] Pende M, Um SH, Mieulet V, Sticker M, Goss VL (2004). S6K1(−/−)/S6K2(−/−) mice exhibit perinatal lethality and rapamycin-sensitive 5'-terminal oligopyrimidine mRNA translation and reveal a mitogen-activated protein kinase-dependent S6 kinase pathway.. Mol Cell Biol.

[44] McNeill H, Craig GM, Bateman JM (2008). Regulation of neurogenesis and epidermal growth factor receptor signaling by the insulin receptor/target of rapamycin pathway in Drosophila.. Genetics.

[45] Orlic M, Spencer CE, Wang L, Gallie BL (2006). Expression analysis of 6p22 genomic gain in retinoblastoma.. Genes Chromosomes Cancer.

[46] Oldham S, Montagne J, Radimerski T, Thomas G, Hafen E (2000). Genetic and biochemical characterization of dTOR, the Drosophila homolog of the target of rapamycin.. Genes Dev.

[47] Tettweiler G, Miron M, Jenkins M, Sonenberg N, Lasko PF (2005). Starvation and oxidative stress resistance in Drosophila are mediated through the eIF4E-binding protein, d4E-BP.. Genes Dev.

[48] Gao X, Zhang Y, Arrazola P, Hino O, Kobayashi T (2002). Tsc tumour suppressor proteins antagonize amino-acid-TOR signalling.. Nat Cell Biol.

