# Supplementary figures and images for: Tuberous Sclerosis Complex 1 Regulates dE2F1 Expression during Development and Cooperates with RBF1 to Control Proliferation and Survival
Source: PLoS Genet. 2010 Aug 19;6(8):e1001071. doi: 10.1371/journal.pgen.1001071 (PMC2924346; doi:10.1371/journal.pgen.1001071)

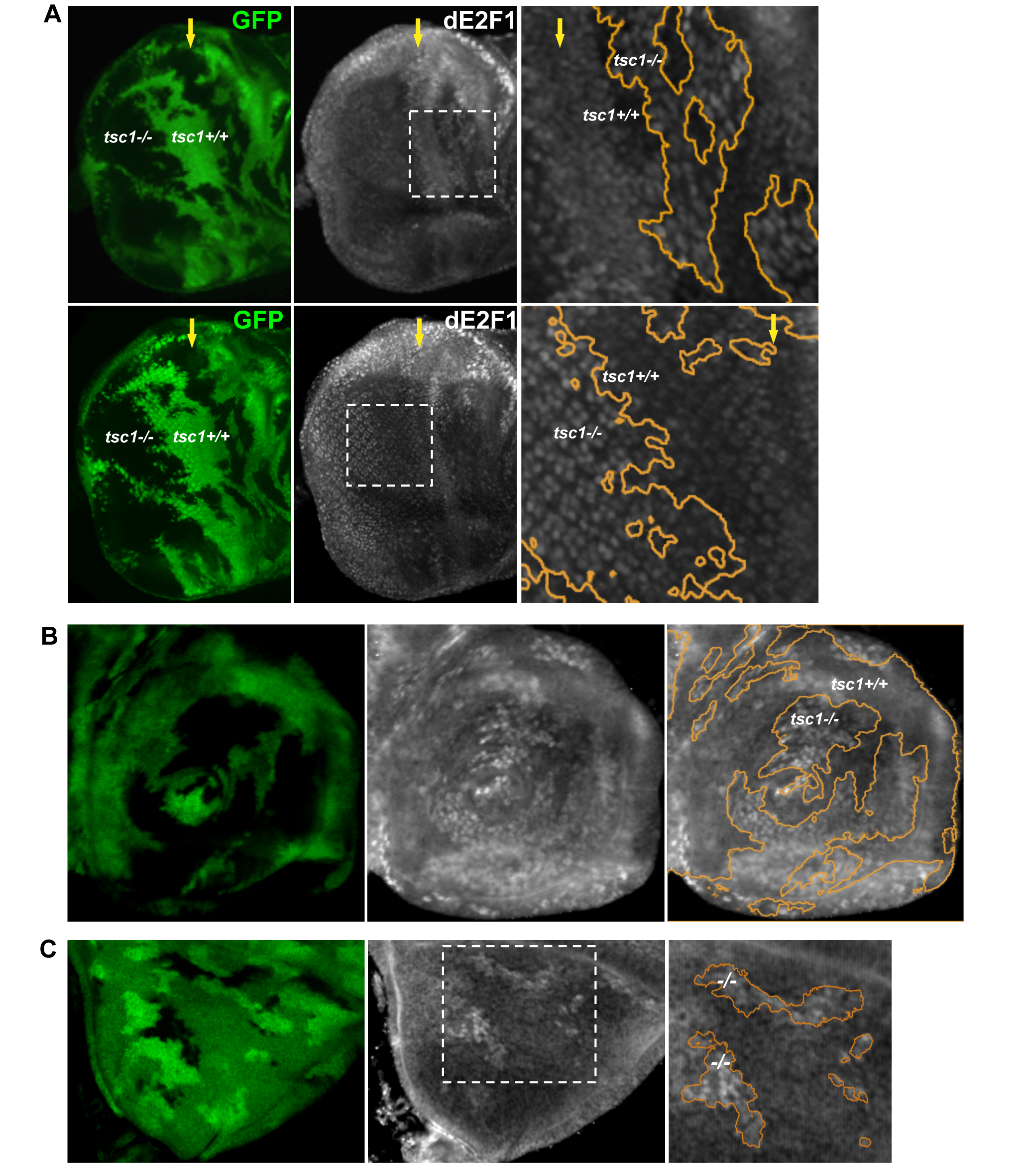

Supplement: Figure S1 — Tsc1 regulates dE2F1 protein level both in proliferating and differentiating cells in imaginal discs. (A) tsc1R453X mutant clones are generated in the eye- antenna disc as previously described and immunostained with an anti-dE2F1 antibody. Images at two different focal planes of a single eye disc are shown. The upper panel shows increased expression of dE2F1 proteins in tsc1 mutant clones at the anterior region of the eye disc. The lower panel shows increased dE2F1 expression in tsc1 mutant clone at the posterior region of the eye disc. The magnified views of indicated area are also presented. (B) An antenna disc that contains tsc1R453X mutant clones is shown. As in eye imaginal discs, dE2F1 expression is increased in tsc1 mutant clones. (C) tsc1R453X mutant clones are generated in the wing disc using heat shock driven Flippase. Presumptive notum area of the wing disc is shown. Note the increased level of dE2F1 proteins in tsc1 mutant clones. (3.18 MB TIF) [file pgen.1001071.s001.tif]

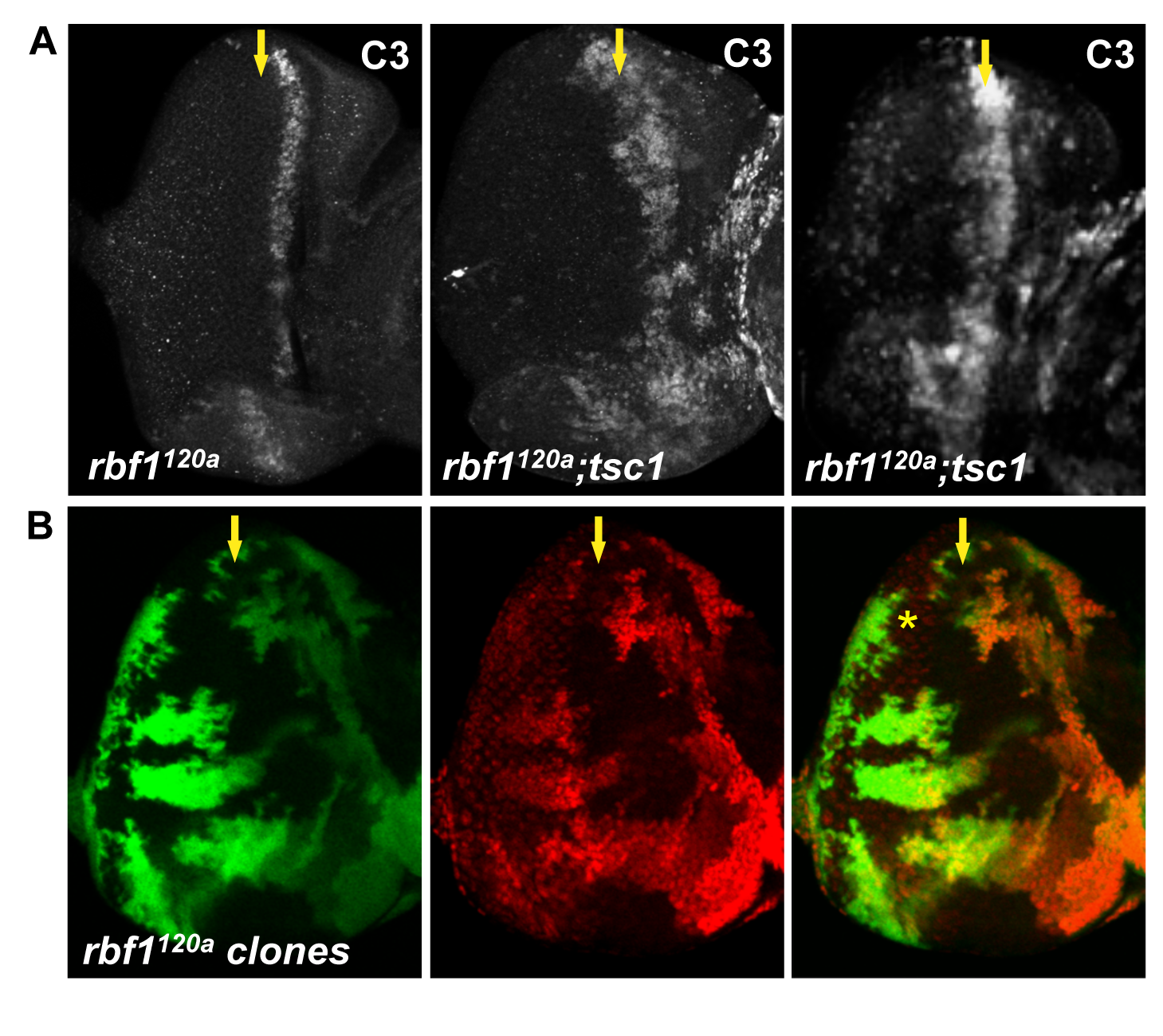

Supplement: Figure S2 — The pattern of ectopic cell death in eye imaginal discs that are mostly composed of rbf1 tsc1 double mutant cells. (A) The patterns of cell death between an rbf1120a eye disc and two eye discs carrying both rbf1120a and tsc1R453X mutations are shown (see Materials and Methods). Apoptotic cells are visualized by the C3 antibody. A dramatic increase in C3 staining is observed at the MF and in the anterior region of the eye discs carrying both rbf1 and tsc1 mutations. (B) rbf1120a mutant clones, marked by absence of GFP, are generated in the eye discs. Note the weak but visible RBF1 staining in rbf1120a mutant clones in the region posterior to the MF (yellow asterisk). (1.38 MB TIF) [file pgen.1001071.s002.tif]

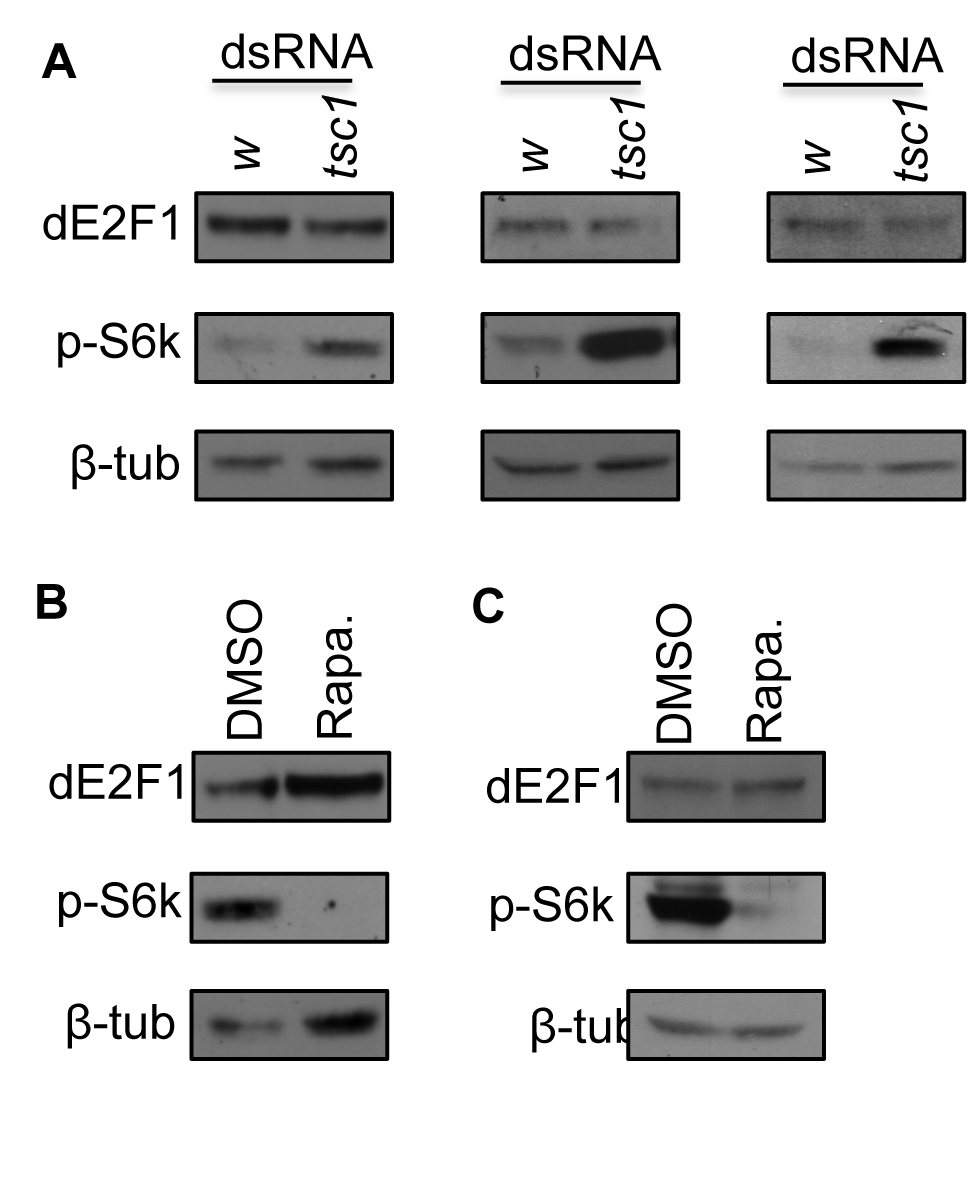

Supplement: Figure S3 — Inactivation of tsc1 nor Tor affects dE2F1 protein level in S2 Drosophila tissue culture cells. (A) S2 cells are treated with either white or tsc1 double strand RNA for 4 days and dE2F1 protein levels are measured by immunoblot. The antibody that recognizes the phospho-specific form of S6k (Cell Signaling, Cat#. 9206) is used to monitor the effect of tsc1 depletion and anti-β-tubulin antibodies are used for loading control. Three independent experimental results are presented. (B) S2 cells are treated with DMSO or DMSO containing Rapamycin (the final concentration of 20 nM). After 16 hours of treatment, dE2F1 protein levels are measured by immunoblot. A phospho-specific S6k antibody is used to monitor the effect of Rapamycin treatment. For each lane, an equal amount of protein extract is loaded 28. (C) S2 cells are treated as described in (B). However, the amount of protein extract loaded in each lane is normalized by cell number and not by protein concentration. Note that S2 cells do not recapitulate the effect observed in imaginal discs. (0.22 MB TIF) [file pgen.1001071.s003.tif]
